# Network analyses of upper and lower airway transcriptomes identify shared mechanisms among children with recurrent wheezing and school-age asthma

**DOI:** 10.3389/fimmu.2023.1087551

**Published:** 2023-01-27

**Authors:** Zhili Wang, Yu He, Qinyuan Li, Yan Zhao, Guangli Zhang, Zhengxiu Luo

**Affiliations:** ^1^ Department of Respiratory Medicine, Children’s Hospital of Chongqing Medical University, Key Laboratory of Child Development and Disorders, National Clinical Research Center for Child Health and Disorders, Ministry of Education, Chongqing, China; ^2^ Chongqing Key Laboratory of Pediatrics, Chongqing, China; ^3^ Department of Respiratory Medicine, Children’s Hospital of Chongqing Medical University, Chongqing, China

**Keywords:** cell deconvolution, gene co-expression network, machine learning, recurrent wheezing, school-age asthma, scRNA-seq, RNA-seq

## Abstract

**Background:**

Predicting which preschool children with recurrent wheezing (RW) will develop school-age asthma (SA) is difficult, highlighting the critical need to clarify the pathogenesis of RW and the mechanistic relationship between RW and SA. Despite shared environmental exposures and genetic determinants, RW and SA are usually studied in isolation. Based on network analysis of nasal and tracheal transcriptomes, we aimed to identify convergent transcriptomic mechanisms in RW and SA.

**Methods:**

RNA-sequencing data from nasal and tracheal brushing samples were acquired from the Gene Expression Omnibus. Combined with single-cell transcriptome data, cell deconvolution was used to infer the composition of 18 cellular components within the airway. Consensus weighted gene co-expression network analysis was performed to identify consensus modules closely related to both RW and SA. Shared pathways underlying consensus modules between RW and SA were explored by enrichment analysis. Hub genes between RW and SA were identified using machine learning strategies and validated using external datasets and quantitative reverse transcription-polymerase chain reaction (qRT-PCR). Finally, the potential value of hub genes in defining RW subsets was determined using nasal and tracheal transcriptome data.

**Results:**

Co-expression network analysis revealed similarities in the transcriptional networks of RW and SA in the upper and lower airways. Cell deconvolution analysis revealed an increase in mast cell fraction but decrease in club cell fraction in both RW and SA airways compared to controls. Consensus network analysis identified two consensus modules highly associated with both RW and SA. Enrichment analysis of the two consensus modules indicated that fatty acid metabolism-related pathways were shared key signals between RW and SA. Furthermore, machine learning strategies identified five hub genes, i.e., CST1, CST2, CST4, POSTN, and NRTK2, with the up-regulated hub genes in RW and SA validated using three independent external datasets and qRT-PCR. The gene signatures of the five hub genes could potentially be used to determine type 2 (T2)-high and T2-low subsets in preschoolers with RW.

**Conclusions:**

These findings improve our understanding of the molecular pathogenesis of RW and provide a rationale for future exploration of the mechanistic relationship between RW and SA.

## Introduction

Wheezing is a common symptom in preschool-age children (2–5 years) and a global public health issue  (1, 2). It is associated with high morbidity and significant health care costs (e.g., 53 million pounds in the UK annually)  (3). Almost 50% of preschoolers experience at least one wheezing episode  (2, 4). Although most preschool children recover from wheezing during the school-age period (aged 6–13 years), some experience recurrent wheezing (RW) and may develop asthma  (2, 5). However, despite substantial efforts, it is difficult to predict which children with RW will develop school-age asthma (SA)  (4, 6), highlighting a critical need to clarify the pathogenesis of RW.

Allergic asthma is the most common type (over 80% of cases) among children and is characterized by a type 2 (T2)-biased airway inflammatory response involving a complex network of epithelial cells, T helper 2 (Th2) cells, group 2 innate lymphoid cells, eosinophils, and mast cells, as well as their major cytokines  (7, 8). Despite recent evidence showing that loss of airway epithelial barrier integrity and impaired wound repair capacity following insults are tightly associated with RW, the biological mechanisms underlying RW remain poorly understood  (9, 10). Previous studies have identified multiple environmental (e.g., atopic sensitization, tobacco exposure, and respiratory tract infections early in life) and genetic risk factors (e.g., chr17q21 locus) shared by RW and SA  (8, 11–13). The intrinsic associations during disease trajectory and similarities in environmental exposures and genetic determinants suggest potential overlap in the mechanisms and pathogenic pathways of RW and SA. Thus, exploring the mechanisms common to RW and SA should increase our understanding of the pathogenesis of RW.

Recent computational methods in systems biology, such as network analysis and machine learning, can facilitate our understanding of disease by analyzing multi-omics data (e.g., RNA-sequencing (RNA-seq)) at the systemic level  (14). Weighted gene co-expression network analysis (WGCNA)  (15) can identify gene modules of highly correlated genes and their association with clinical or phenotypic traits. WGCNA also applies a unique consensus network-based approach  (16) to reveal consensus gene modules shared among datasets and consensus modules related to clinical trait information. To the best of our knowledge, previous studies have not explored the molecular networks shared by RW and SA.

High-throughput single-cell RNA sequencing (scRNA-seq) enables comprehensive analysis of tissue microenvironments  (17). Although scRNA-seq is a powerful tool for resolving cellular heterogeneity, it remains impractical for large-scale analysis  (18). The recently developed CIBERSORTx algorithm allows deconvolution of bulk RNA-seq data to estimate the abundances of member cell types in a mixed cell population using signature genes derived from scRNA-seq for large-scale tissue dissection  (19). However, our current understanding of the changes in the RW airway microenvironment remains limited.

As RW affects the entire airway, integrated study of the upper (e.g., nasal) and lower (e.g., tracheal) airways is a powerful approach for understanding RW  (20). Here, based on nasal and tracheal bulk RNA-seq data, scRNA-seq analysis, cell deconvolution (using CIBERSORTx), and consensus network analysis, we identified consensus gene modules and critical cellular components shared by RW and SA. Molecular pathways common to RW and SA were explored by enrichment analysis of key consensus modules. Hub genes between RW and SA were identified using machine learning strategies, then validated using independent external datasets and quantitative reverse transcription-polymerase chain reaction (qRT-PCR). The potential value of hub genes in defining RW subsets was also determined using nasal and tracheal RNA-seq data. The identified hub genes, cellular components, and pathways between RW and SA should provide new insights into the pathogenesis of RW and help identify RW-affected preschoolers with distinct molecular mechanisms of airway inflammation.

## Materials and methods

### Ethics statement

This study was approved by the Ethics Committee of the Children’s Hospital of Chongqing Medical University. Written informed consent was obtained from the legal guardians of the study participants before enrollment.

### Patient recruitment

A total of 32 preschoolers with RW (defined as ≥ 3 physician-diagnosed wheezing episodes)  (21, 22), 15 children with SA  (23), and 18 control individuals (patients without current respiratory tract infection, current wheezing, or history of allergy or wheezing) undergoing bronchoscopy were enrolled in the study. Collection of bronchoalveolar lavage fluid (BALF) from controls and patients with RW was carried out using standard procedures  (24). BALF was gently aspirated and centrifuged at 2 500 rpm for 5 min at 4°C after collection. The bronchoalveolar lavage (BAL) cells were resuspended in phosphate-buffered saline (PBS) and stored at −80°C. Details on subject characteristics are included in **Supplementary File 1**; **Table S1**.

### RNA-seq dataset collection and processing

RNA-seq data (raw count matrix) of RW, SA, and healthy controls were obtained from the Gene Expression Omnibus (GEO) database at the National Center for Biotechnology Information (NCBI; accession number GSE118761). This dataset included nasal and tracheal brushing samples from RW (n = 14), SA (n = 13), and healthy (n = 14) groups (**Supplementary File 1**; **Table S2**). To prevent sampling noise caused by lowly expressed genes, genes with low expression (< 30 counts in total across all nasal or tracheal samples) were excluded. For subsequent analysis and visualization of count data, variance stabilizing transformation (VST) normalized expression values were calculated using the “DESeq2”  (25) R package (v1.34.0).

### Identification and analysis of differentially expressed genes

The DEGs between groups (RW or SA vs. control) were identified using the “DESeq2” R package (v1.34.0), with *P* < 0.05 and |log2fold-change| > 0.5 considered significant. To identify common DEGs (co-DEGs) between RW and SA in the upper and lower airways, the “UpSetR” (26) R package (v1.4.0) was used to construct an UpSet diagram.

### scRNA-seq data acquisition and processing

The scRNA-seq data (count matrix) of airway samples collected from 18 healthy children were obtained from the FigShare repository (**Supplementary File 1**; **Table S2**). The R package Seurat  (27) (v4.1.2) was used to process the scRNA-seq data. To ensure high-quality single cells were used for downstream analysis, cells expressing fewer than 500 genes and cells with more than 15% mitochondrial reads were filtered out. In total, 38 399 filtered cells were used for further analysis.

Raw data were normalized using the “NormalizeData” function, and 2 500 highly variable genes were identified using “FindVariableFeatures”. Principal component analysis (PCA) was then performed for dimensionality reduction after data scaling. The top 40 principal components were selected for downstream analysis. The Uniform Manifold Approximation and Projection (UMAP) algorithm was used for cell visualization. The “FindClusters” function was used for cell clustering. To annotate cell clusters, the DEGs for each cell cluster were identified by comparing each cluster to all other clusters with the “FindAllMarkers” function. Genes with adjusted *P* < 0.05 were considered DEGs. The cell subsets were annotated based on the DEGs and known markers  (28).

### Cellular composition in upper and lower airways based on CIBERSORTx

The CIBERSORTx online platform (https://cibersortx.stanford.edu/) was applied to infer the cellular composition of the bulk airway transcriptomes. We first prepared and uploaded the single-cell expression matrix from our scRNA-seq analysis according to the CIBERSORTx instructions using default parameters. We ran “CIBERSORTx” and obtained a signature matrix of 18 cell types, then uploaded the gene expression matrix data for the RW, SA, and control groups. The previously obtained signature matrix was used for deconvolution analysis, with all parameters set to default. After running “CIBERSORTx”, we obtained the relative proportions of the 18 cell subsets in each sample.

### Consensus WGCNA

WGCNA was performed using the “WGCNA”  (15) R package (v4.1.2) and the VST-normalized gene expression profile data were used for network construction. We first constructed nasal and tracheal co-expression gene networks for RW and SA in parallel using default parameters. The RW modules were then correlated with the SA modules (**Supplementary File 1**; **Figures S1A, B**). We calculated the overlap of each pair of RW-SA modules and used Fisher’s exact test to assign a *P-*value to each pairwise overlap. Next, we built nasal and tracheal consensus networks for RW and SA, respectively. Based on scale-free topology criteria, we selected appropriate soft-threshold power values for network construction (**Supplementary File 1**; **Figures S2**, **S3**). The Dynamic Tree Cut method was used to identify different modules, with modules showing similar expression patterns then merged (**Supplementary File 1**; **Figures S4A, B**). The minimum module size was set to 40 and the DeepSplit parameter was set to 2.5.

### Identification of key consensus modules shared by RW and SA

In order to identify consensus modules significantly correlated with clinical features of RW and SA (and in the same positive or negative direction), we performed Pearson correlation analysis of consensus module eigengenes and clinical traits (atopy and cellular compositions inferred from CIBERSORTx). Modules showing significant positive correlations with both RW and SA, as well as important phenotypic traits, were considered as key consensus modules.

### Functional annotation and pathway enrichment analysis

To identify the biological functions of the co-DEGs and genes within the key consensus modules, Gene Ontology (GO) (29) and Kyoto Encyclopedia of Genes and Genomes (KEGG)  (30) pathway analyses were performed using the “clusterProfiler” R package (v4.2.2), with significant enrichment considered at *P* < 0.05.

### Screening of hub genes common to RW and SA based on machine learning

Two machine learning algorithms, Random Forest (RF)  (31) and support vector machine-recursive feature elimination (SVM-RFE)  (32), were used to screen hub genes (i.e., genes within key consensus modules) highly correlated with RW and SA. Specifically, RF and SVM-RFE were used to estimate how well each candidate hub gene correctly classified RW or SA compared to the controls, and to detect the number of feature genes required to separate groups with maximum accuracy. The RF and SVM-RFE modeling procedures were based on three-fold cross-validation (CV) using the “randomForest” and “e1071” R packages, respectively. The classification accuracies of different numbers of feature genes were determined for RF and SVM-RFE, with those showing the highest classification accuracy retained to determine the final hub genes. Hub genes were selected by intersecting the co-DEGs and common feature genes identified by RF and SVM-RFE.

For testing hub gene efficacy, receiver operating characteristic (ROC) curves and corresponding areas under the ROC curves (AUC) were calculated for each hub gene based on their standardized expression levels.

### External validation of hub gene expression and convergent mechanisms in RW and SA

To validate the expression profiles of the hub genes in RW and SA, external validation was performed with two independent SA nasal gene expression datasets (GSE19187 and GSE65204) and one RW nasal gene expression dataset (GSE103166). The GSE103166 and GSE65204 datasets were used to validate shared mechanisms between RW and SA. The GSE103166 dataset contained 56 RW and 21 healthy individuals, the GSE65204 dataset contained 36 SA and 33 healthy individuals, and the GSE19187 dataset contained 13 SA and 11 healthy individuals. Detailed information on these datasets is provided in **Supplementary File 1**; **Table S2**.

### RNA extraction and qRT-PCR for hub genes

Total RNA was extracted from BAL cells using TRIzol reagent (Invitrogen, USA), and purified using a Micro Total RNA Extraction Kit (Tianmo Biotech, China). cDNA was synthesized using a PrimeScript RT Kit (TaKaRa, Japan) according to the manufacturer’s instructions. Reactions were carried out in a total volume of 10 μL, including 5 μL of TB Green^®^Premix Ex Taq™ II (TaKaRa, Japan), 0.2 μL of each specific primer, 2.6 μL of ddH_2_O, and 2 μL of cDNA. The relative expression levels of the hub genes were calculated using the2^-ΔΔCt^ method. GAPDH was used as an internal reference. The specific primers for each gene are provided in **Supplementary File 1**; **Table S3**.

### Transcriptional regulatory network of hub genes

To identify the potential regulatory transcription factors (TFs) for the hub genes, we performed enrichment analysis of TF binding motifs (TFBMs) and TFs surrounding the transcription start site (TSS) of genes using the “RcisTarget”  (33) R package (v1.14.0). Significantly enriched TFBMs (normalized enrichment score (NES) > 3.0) were annotated to TFs using the provided annotation database. The TF-target network was visualized with Cytoscape (v3.8.2)  (34).

### Statistical analysis

**Figure 1:**
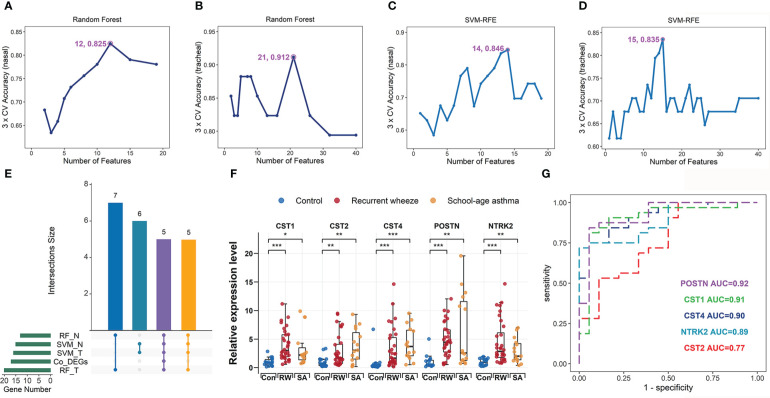
Identification and validation of hub genes shared by RW and SA. Process of identifying hub genes using random forest (RF) algorithm across nasal **(A)** and tracheal samples **(B)**. Identifying hub genes based on support vector machine-recursive feature elimination (SVM-RFE) across nasal **(C)** and tracheal samples **(D)**. X-axis denotes number of feature genes; y-axis represents classification accuracy. Annotated numbers represent number of feature genes corresponding to maximum classification accuracy. **(E)** UpSet diagram showing 16 overlapping hub genes between co-DEGs and candidate hub genes screened by two machine learning algorithms. **(F)** qRT-PCR validation of POSTN, CST1, CST2, CST4, and NTRK2 expression in bronchoalveolar lavage (BAL) cells from controls, patients with SA, and preschoolers with RW. Statistical significance was assessed using Wilcoxon rank-sum test. Asterisks indicate *P*-values for RW or SA versus control. *P < 0.05, **P < 0.01, ***P < 0.001. **(G)** ROC curves evaluating discriminatory power of hub genes for children with RW. AUC, area under ROC curve; co-DEGs, common differentially expressed genes; con, control; N, nasal; qRT-PCR, quantitative reverse-transcription polymerase chain reaction; ROC, receiver operating characteristic; RW, recurrent wheezing; T, tracheal.

**Figure 2:**
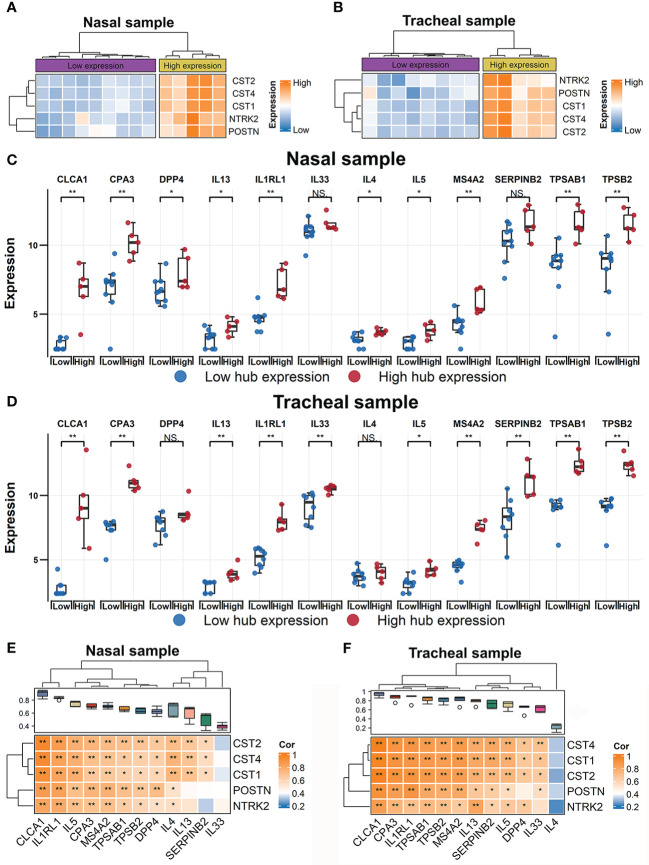
Identification of RW patient subsets using expression profiles of hub genes. Heatmap of hierarchical clustering of POSTN, CST1, CST2, CST4, and NTRK2 expression levels across all subjects with RW in nasal **(A)** and tracheal samples **(B)**, respectively. Comparison of gene expression levels of 12 T2 inflammatory markers between high- and low-hub expression groups of all RW patients across nasal **(C)** and tracheal samples **(D)**. Statistical significance was assessed using Wilcoxon rank-sum test. Asterisks indicate *P*-values for hub high-expression versus hub low-expression. *P < 0.05, **P < 0.01. Correlation heatmaps showing associations between hub genes (row) and 12 T2 inflammatory markers (column) across nasal **(E)** and tracheal samples **(F)**. Boxplot above heatmap showing correlation coefficients obtained by Pearson correlation analyses between each T2 inflammatory genes and hub genes. Blue to orange gradient coloration implies increased Pearson correlation coefficient. Correlation coefficients (r) and *P*-values were obtained by Pearson correlation analysis. *P < 0.05, **P < 0.01. NS, no significance; RW, recurrent wheezing; T2, type 2.

**Figure 3:**
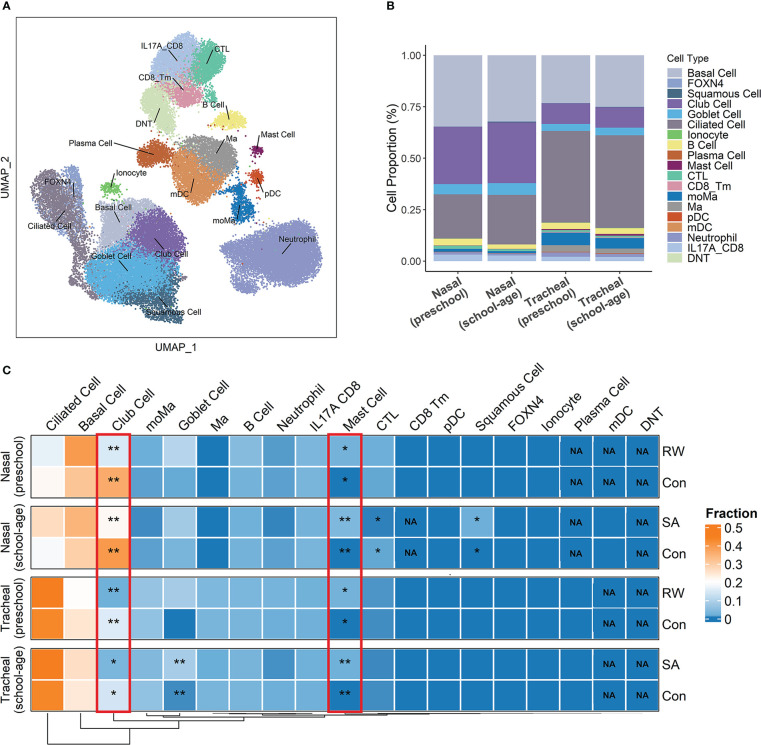
Deconvolution of airway transcriptome data to infer critical cell components shared by RW and SA. **(A)** Overall cell-type composition of 38 399 cells from 18 healthy children visualized using UMAP. CD8_Tm, memory CD8+ T cells; CTL, cytotoxic T cells; DNT, double-negative T cells; FOXN4, FOXN4+ cells; IL-17A_CD8, IL-17A-expressing CD8+ T cells; mDC, myeloid dendritic cells; Ma, macrophages; moMa, monocyte-derived macrophages; pDCs, plasmacytoid dendritic cells. **(B)** Histogram displaying proportion of each cell type in preschool and school-age subjects across nasal and tracheal samples. **(C)** Heatmap of proportion of each cell type across upper and lower airways of RW, SA and healthy controls inferred by cell deconvolution analysis. NA indicates *P-*value could not be calculated because corresponding cellular components were not detected by CIBERSORTx. Statistical significance was assessed using Wilcoxon rank-sum test. Asterisks indicate *P*-values for RW or SA versus control. *P < 0.05, **P < 0.01. Con, control; NA, not applicable; RW, recurrent wheezing; SA, school-age asthma.

All continuous data are expressed as mean ± standard deviation (SD). All statistical analyses were conducted using R (v4.1.2; https://www.r-project.org/). Wilcoxon’s rank-sum test was used to compare gene expression levels (**Figures 1F**, **2C, D** and **Supplementary File 1**; **Figures S9E–G**) and cellular compositions inferred by CIBERSORTx between groups (**Figure 3C**). Pearson correlation analysis (**Figures 2E, F**) was performed to obtain correlation coefficients (r) and *P*-values. A *P*-value of < 0.05 was considered statistically significant.

## Results

### Identification of co-DEGs between RW and SA in upper and lower airways

**Figure 4:**
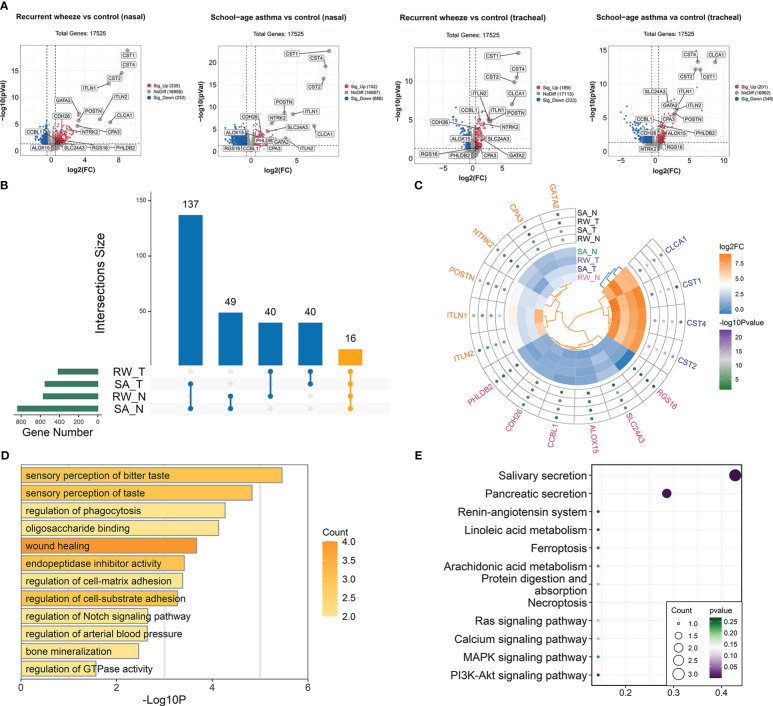
Identification of co-DEGs shared by RW and SA across upper and lower airways. **(A)** Volcano plots showing DEGs between healthy controls and children with RW/SA. **(B)** UpSet diagram showing overlapping DEGs between RW and SA across nasal and tracheal samples. In total, 16 co-DEGs were shared by RW and SA across nasal and tracheal samples. **(C)** Heatmap visualization of differential expression changes (colors in inner circle indicates corresponding log2FC) and significance values (colors in outer circle represent corresponding -log10*P*-value) for 16 co-DEGs derived from integrated analysis. **(D)** GO term enrichment analysis of co-DEGs. **(E)** KEGG pathway enrichment analysis of co-DEGs. co-DEGs, common DEGs; DEGs, differentially expressed genes; FC, fold-change; GO, Gene Ontology; KEGG, Kyoto Encyclopedia of Genes and Genomes; N, nasal; RW, recurrent wheezing; SA, school-age asthma; T, tracheal.

Compared with the healthy controls, we identified 567 DEGs (335 up-regulated and 232 down-regulated) and 549 DEGs (201 up-regulated and 348 down-regulated) in the RW nasal and tracheal samples, respectively, and 838 DEGs (152 up-regulated and 686 down-regulated) and 412 DEGs (189 up-regulated and 223 down-regulated) in the SA nasal and tracheal samples, respectively (**Figure 4A**). The UpSet diagram in **Figure 4B** shows the number of overlapping DEGs between RW and SA across nasal and tracheal samples. In total, 16 co-DEGs were shared between RW and SA across the upper and lower airways (**Figures 4A, C**). Compared to the healthy controls, these genes were overrepresented in RW and SA. The co-DEGs were retained for identifying hub genes shared by RW and SA.

To investigate the biological behaviors of the co-DEGs, GO functional annotation and KEGG enrichment analysis were performed. Results indicated that the co-DEGs were mainly involved in sensory perception of taste, wound healing, endopeptidase inhibitor activity, and regulation of cell-matrix adhesion (**Figure 4D**). Salivary secretion and pancreatic secretion were the major biological pathways involved (**Figure 4E**).

### Inferring critical cell components shared by RW and SA through deconvolution of airway transcriptome data

To understand changes in the RW and SA airway microenvironments as well as potential critical cell populations shared by RW and SA, we performed cell deconvolution of the upper and lower bulk transcriptome data to deduce changes in cell-type frequency within the airways of RW and SA.

First, we analyzed the scRNA-seq data using cells from airway samples of 18 healthy children. After stringent quality control, 38 399 cells were retained for further analysis. Unsupervised clustering by UMAP identified 18 cell subsets (**Figure 3A**) based on representative marker genes from previous research   (28). The cell clusters identified by cell lineage-specific marker gene expression are shown in **Supplementary File 1**; **Figure S5**. Based on the CIBERSORTx algorithm, a signature matrix of 2 360 genes in 18 cell clusters was created (**Supplementary File 2**; **Table S4**).

Next, using the signature matrix derived from scRNA-seq analysis, we calculated the relative proportions of the 18 cell subsets for the RW, SA, and control samples using CIBERSORTx. The histogram in **Figure 3B** shows the proportions of each cell type in the upper and lower airways of preschool and school-age children. We then examined fractional differences in each cell type between RW and SA and the control group. Interestingly, a higher fraction of mast cells and lower fraction of club cells were observed in both the upper and lower airways of the RW and SA patients compared to the healthy controls (**Figure 3C**).

### Identification of key consensus gene modules shared by RW and SA

To explore the possibility that RW and SA share certain mechanisms and biological pathways, we analyzed similarities in their gene expression networks across nasal and tracheal transcriptomes using WGCNA. WGCNA can identify gene modules with similar gene expression patterns, thus providing biological insight based on the principal that genes with highly correlated expression patterns are likely to participate in the same biological processes. By constructing co-expression gene networks for RW and SA in parallel, we identified 25 and 25 network modules for RW and SA from their nasal transcriptomes, respectively (**Supplementary File 1**; **Figure S1A**), and 26 and 25 network modules for RW and SA from their tracheal transcriptomes, respectively (**Supplementary File 1**; **Figure S1B**). To determine whether network modules identified in RW could also be identified in SA, we explored the correlations between RW-specific and SA-specific modules. Results showed that most RW-specific modules had one or more SA module counterparts in both the upper and lower airways (**Supplementary File 1**; **Figures S1A, B**), thus suggesting possible similarities in the underlying transcriptional networks between RW and SA.

**Figure 5:**
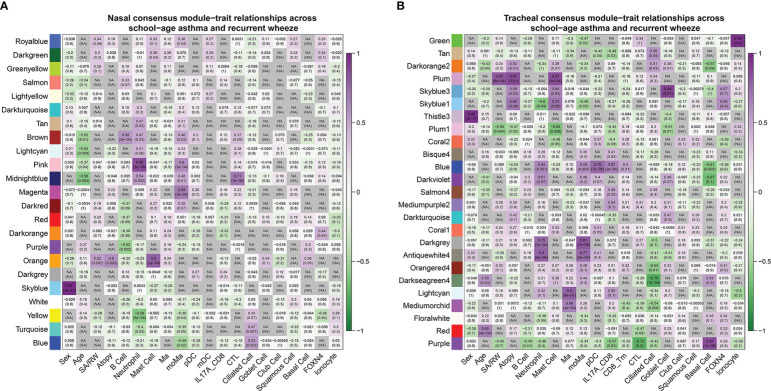
Consensus module-trait relationships across RW and SA in nasal **(A, B)** tracheal networks. Each module is represented by its eigengene value and Pearson correlation analysis between eigengene values and clinical traits was performed. Each color represents one consensus gene module. Each row corresponds to a module eigengene, and each column to a clinical trait. Each cell contains corresponding Pearson correlation coefficient (first number) and *P*-value (number in parenthesis). Green to purple gradient coloration implies increased Pearson correlation coefficient. Nasal orange module and tracheal plum module were significantly positively associated with RW, SA, atopy, and mast cells. RW, recurrent wheezing; SA, school-age asthma.

Next, we built consensus networks using the nasal and tracheal transcriptome data, with the resulting network modules representing robust gene co-expression patterns shared by RW and SA. The nasal and tracheal consensus networks contained 23 and 25 modules of co-expressed genes, respectively (**Supplementary File 1**; **Figures S4A, B**). To determine key consensus network modules highly correlated with both RW and SA, we performed Pearson correlation analysis between consensus module eigengenes and clinical traits of RW and SA, including the fractions of the 18 cell subsets inferred by CIBERSORTx. Heatmaps were constructed to show the relationship between nasal (**Supplementary File 1**; **Figures S6A, B**) and tracheal (**Supplementary File 1**; **Figures S7A, B**) consensus modules and clinical trait information for RW and SA. To obtain consensus module-trait relationships across both RW and SA, we integrated the separate consensus module-trait relationships specific to RW and SA. We retained the lower absolute value in the two sets with the same correlation sign, and “NA” for those with the opposite trend. **Figures 5A, B** shows the integrated consensus module-trait relationships in both RW and SA. Of note, in the upper and lower airway consensus networks, only two consensus modules (orange module in the nasal consensus network and plum module in the tracheal consensus network) were significantly positively correlated with RW and SA. Interestingly, these two consensus modules were also significantly positively correlated with atopy and mast cells. Therefore, the nasal orange module and tracheal plum module were selected as key consensus gene modules shared by RW and SA.

### Key consensus module gene signatures can distinguish RW and SA from controls across upper and lower airways

**Figure 6:**
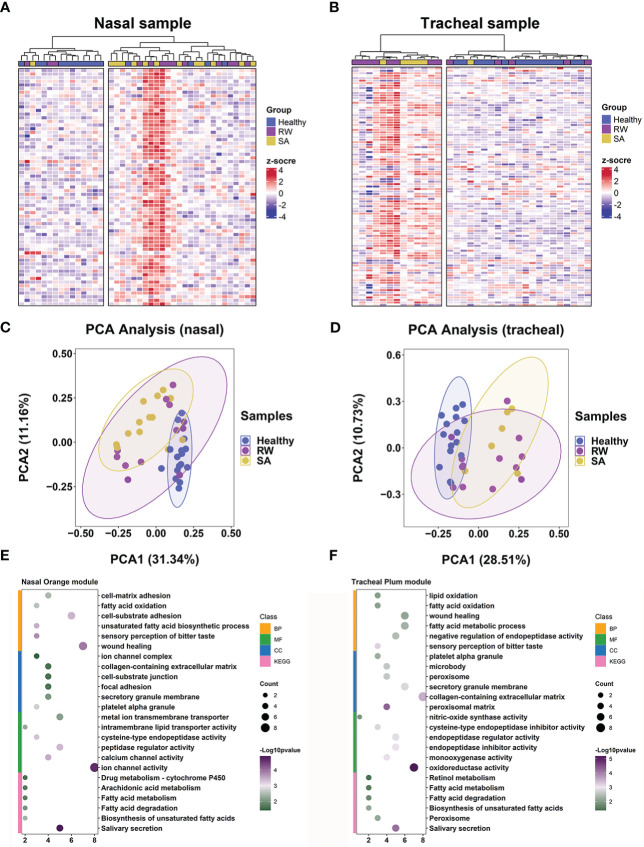
Key consensus module gene signatures can distinguish RW and SA from controls across both upper and lower airway samples. Heatmaps of expression patterns of 66 genes in nasal orange consensus module across all nasal samples **(A)** and 98 genes in tracheal plum consensus module across all tracheal samples **(B)**, where genes and samples were ranked using hierarchical clustering. PCA across all nasal samples based on 66 genes in nasal orange module **(C)** and across all tracheal samples based on 98 genes in tracheal plum module **(D)**. Dot plot showing enriched GO terms (BP, CC, and MF) and KEGG pathways for nasal orange module **(E)** and tracheal plum module **(F)**, respectively. BP, biological process; CC, cellular component; KEGG, Kyoto Encyclopedia of Genes and Genomes; MF, molecular function; PCA, principal component analysis; RW, recurrent wheezing; SA, school-age asthma.

Next, we explored whether key consensus module gene signatures could be distinguished between children with RW and SA and healthy subjects. We performed hierarchical clustering analyses using the 66 (**Supplementary File 3**; **Table S5**) and 98 (**Supplementary File 4**; **Table S6**) genes in the nasal orange module and tracheal plum module, respectively. Heatmap visualization of the genes in the nasal orange module (**Figure 6A**) and tracheal plum module (**Figure 6B**) indicated that: (1) genes were more highly expressed in the RW and SA individuals; (2) hierarchical clustering grouped samples into two major clusters, with RW and SA clustered together based on similar expression patterns; and (3) hierarchical clustering using key consensus module gene signatures clearly distinguished RW and SA patients from the controls. Furthermore, PCA of the genes in the nasal orange module and tracheal plum module also distinguished RW and SA patients from the controls in both the nasal and tracheal samples (**Figures 6C, D**). Notably, the RW and SA individuals tended to cluster together. These results suggest the potential biological significance of the co-expressed genes in the key consensus modules and highlight the possibility of shared underlying molecular mechanisms between RW and SA.

### Identification and validation of convergent mechanisms in RW and SA

We further explored shared mechanisms between RW and SA by identifying significantly overrepresented biological pathways and GO terms for the two key consensus modules. Overall, the nasal and tracheal consensus modules were associated with several similar GO terms, including fatty acid oxidation, fatty acid metabolic process, and sensory perception of bitter taste (**Figures 6E, F**). Fatty acid metabolism-related pathways were also significantly enriched in both the nasal orange and tracheal plum modules (**Figures 6E, F**).

To validate the convergent mechanisms in RW and SA, we performed similar analysis using two larger validation datasets, GSE103166 (56 RW patients and 21 controls) and GSE65204 (36 SA patients and 33 controls). As expected, fatty acid metabolism-related biological processes and pathways were significantly enriched for both RW and SA (**Supplementary File 1**; **Figure S8**).

### Identification and validation of hub genes shared by RW and SA

To further screen hub genes most relevant to RW and SA within the key consensus modules, we applied the RF and SVM-RFE machine learning algorithms. Gene expression profiles in the nasal orange module (66 genes) and tracheal plum module (98 genes) were extracted, and the RF and SVM-RFE algorithms were run. Twelve and 14 feature genes in the nasal orange module were identified with the best classification accuracy based on RF and SVM-RFE, respectively (**Figures 1A, C**), while 21 and 15 feature genes in the tracheal plum module were identified with the best classification accuracy based on RF and SVM-RFE, respectively (**Figures 1B, D**). As shown in the UpSet diagram in **Figure 1E**, five genes (CST1, CST2, CST4, POSTN, and NTRK2) overlapped between the two algorithms. These five genes were also co-DEGs between RW and SA (**Figure 4C**) and were therefore selected as hub genes.

To verify the efficacy of the hub genes, ROC analyses were performed to evaluate the power of each hub gene to distinguish RW and SA patients from healthy controls. Overall, the results showed that each hub gene displayed a moderate ability to distinguish children with RW and SA from healthy controls across both nasal and tracheal samples (**Supplementary File 1**; **Figures S9A–D**).

To further validate transcriptome data accuracy, we analyzed the expression levels of the hub genes using two independent SA gene expression datasets (GSE19187 and GSE65204) and one RW gene expression dataset (GSE103166). As expected, all five genes were significantly up-regulated in both RW and SA patients compared to the controls (**Supplementary File 1**; **Figures S9E–G**). Next, we examined the mRNA expression levels of hub genes using qRT-PCR of BAL samples from 18 controls, 15 children with SA, and 32 preschoolers with RW. Similar results were obtained after comparing the expression levels of hub genes, revealing that CST1, CST2, CST4, POSTN, and NTRK2 were highly overrepresented in preschoolers with RW and patients with SA (**Figure 1F**). Furthermore, CST1, CST2, CST4, and NTRK2 displayed moderate-to-high discriminatory power (AUC = 0.91, 0.77, 0.90, and 0.89, respectively) and POSTN showed high discriminatory power (AUC = 0.92) for distinguishing RW from controls (**Figure 1G**).

We next performed enrichment analysis of TFs to identify potential upstream regulators of the hub genes. All enriched TFs and their corresponding AUC and NES values are shown in **Supplementary File 5**; **Table S7**. The TF-gene interaction networks of the top 10 enriched TFs for each hub gene and corresponding hub genes are shown in **Supplementary File 1**; **Figure S10**. Results indicated that the master TF regulator of CST1, CST2, and CST4 was TRIM28, which had the highest NES, while the main TF regulator for POSTN and NTRK2 was TBPL2.

### Hub gene expression profiles could be used to identify RW patient subsets

Previous studies in adults have shown that POSTN and several other genes can be used to define subsets with T2-high and T2-low asthma  (35–37). Thus, we explored whether the combined expression signatures of the five hub genes could be used to classify RW subjects into different subsets. Using the nasal and tracheal transcriptomes, we performed hierarchical clustering analysis of all subjects with RW based on the expression levels of POSTN, CST1, CST2, CST4, and NTRK2. Approximately 30% of RW patients with high hub gene expression were grouped together in one major branch of the dendrogram in both the nasal (**Figure 2A**) and tracheal samples (**Figure 2B**). We then compared the expression levels of 12 T2 inflammatory markers  (35, 38) between RW patients with high and low hub gene expression. Interestingly, most T2 marker genes (e.g., interleukin-5 (IL-5), IL-13, and IL1RL1) showed significantly higher expression in subjects with high hub gene expression than in those with low hub gene expression (**Figures 2C, D**). Furthermore, we found high positive correlations between the expression levels of hub genes and T2 inflammatory genes (**Figures 2E, F**). Taken together, these results suggest that the identified hub genes could potentially be used for defining subsets (T2-high vs. T2-low) of preschoolers with RW.

## Discussion

To the best of our knowledge, this is the first study to investigate the potential common mechanisms underlying RW and SA using cell deconvolution and network analysis of nasal and tracheal transcriptomes. Our results support the possibility of overlapping mechanisms based on the similarities in transcriptional networks between RW and SA in both the upper and lower airway samples. Cell deconvolution analysis indicated that mast cells and club cells were critical cellular components shared by RW and SA. Furthermore, consensus network analysis identified key nasal and tracheal co-expressed gene modules shared by RW and SA, functionally associated with fatty acid metabolism-related biological processes and pathways. We also identified five hub genes (POSTN, CST1, CST2, CST4, and NTRK2) highly associated with RW and SA, with mRNA expression validated using external datasets and qRT-PCR. Finally, we found that the gene signature of the five hub genes may be used to determine T2-high and T2-low subsets of preschoolers with RW.

Using cell deconvolution, we showed that the proportion of mast cells increased, and the proportion of club cells decreased in both the RW and SA airways. Mast cells are key factors in acute allergic reactions in sensitized asthmatic patients. When two adjacent immunoglobulin E (IgE) molecules are cross-linked by an allergen, mast cells are activated to release biologically active mediators such as histamine and neutral proteases such as tryptase and chymase  (39, 40). They also produce lipid mediators and T2-associated cytokines (IL-4, IL-5, IL-9, and IL-13), thereby reinforcing the T2 environment  (39, 40). However, little is known about the changes and functional significance of mast cells in RW, and further research is needed to explore their precise functional roles. Airway epithelial injury and epithelial barrier dysfunction play key roles in the development and progression of asthma  (41, 42). Epithelial damage has been observed in asthmatic and wheezy pediatric airways, and dysregulated repair following insult has been implicated in RW and SA pathogenesis  (9, 10, 43). Club cells of the small airways secrete a specific secretoglobin family 1A member 1 protein (SCGB1A1)  (44). In response to epithelial injury, club cells differentiate into ciliated and mucus-secreting goblet cells to restore epithelial integrity  (45). SCGB1A1-positive epithelial cells are significantly lower in the small airways of adult asthmatic subjects with significantly decreased BALF and serum SCGB1A1 levels compared to controls  (46, 47). In addition, mutations in the SCGB1A1 gene are associated with an increased risk of childhood asthma and a significant decrease in serum concentrations of SCGB1A1  (48, 49). Although these findings suggest that club cells may play important roles in epithelial repair after injury in RW and SA, their contributions to disease pathogenesis are not well characterized, which will be of interest in future investigations. Here, cell deconvolution analysis yielded mixed cell populations, with more than 80% (84%–92%) of cells identified as epithelial cells in the upper and lower airways (**Figure 3B**). Nasal and bronchial brushings generally produce over 90% epithelial cell populations  (50), underscoring the robustness of our deconvolution analysis. However, the presence of several minor immune cells and relatively scarce cells in the mixed cell populations may make the cell composition estimates less accurate. Future studies, especially scRNA-seq studies focusing on RW and SA, are necessary for more precise exploration of cellular heterogeneity within the complex inflammatory airway microenvironments.

We compared the gene co-expression networks in RW and SA through WGCNA and found high similarities (**Supplementary File 1**; **Figure S1**), suggesting common underlying mechanisms between RW and SA. Subsequently, using consensus network analysis, the nasal orange and tracheal plum consensus modules were shown to be significantly correlated with both RW and SA. Interestingly, the two modules were also positively correlated with atopy and mast cells. Associations between atopic history in early life and increased risk of asthma and responsiveness to bronchial allergen challenge have been reported in many pediatric cohorts  (51, 52). Therefore, the co-expressed genes in both modules may represent functionally important transcriptional changes associated with the initiation and progression of RW and SA. We also noticed that the tracheal plum1 consensus module was significantly negatively correlated with both RW and SA, as well as atopy and mast cells (**Figure 5B**), while no corresponding nasal consensus module was significantly negatively correlated with RW and SA (**Figure 5A**). This is perhaps not surprising. Although most nasal consensus modules significantly correlated with clinical traits of RW and SA had one or more tracheal consensus module counterparts (showing the same sign), consistent with the “united airways concept”  (53) (i.e., under disease settings, a pathological process in one region of the airway would affect the function of the entire airway), the gene network modules specific to nasal or tracheal regions reflected region-specific patterns of regulation.

We also investigated the possible shared mechanisms between RW and SA by exploring the biological pathways behind the nasal orange and tracheal plum consensus modules. We found that fatty acid metabolism-associated biological processes and pathways, such as fatty acid oxidation and degradation, were the most common and were overrepresented in both modules (**Figures 6E, F**). Fatty acids are key structural components of phospholipids, and the role of fatty acid metabolites generated through the arachidonic acid (AA) pathway in asthma has long been appreciated  (54). Fatty acid catabolism produces leukotrienes (LTs) and prostaglandins (PGs), which can be derived from AA, a polyunsaturated fatty acid present in cell membrane phospholipids  (54). Allergen exposure results in the secretion of phospholipase A2, which is responsible for the release of AA from cell membranes  (54). LTs play multiple roles in the pathophysiology of asthma by inducing bronchoconstriction, recruiting inflammatory cells, inducing plasma extravasation, and driving tissue edema  (55). PGs exert complex biological effects on the pathophysiological processes of asthma by binding to one or more PG-specific receptors. For instance, prostaglandin D2 (PGD2) activates Th2 lymphocytes by binding to PGD2 receptor 2, inducing eosinophil and Th2 cell chemotaxis to the site of allergic inflammation  (56); as an endogenous counterpart to pro-inflammatory mediators, prostaglandin E2 (PGE2) protects against allergic responses and airway inflammation by inhibiting the functions of eosinophils and macrophages  (57). Given the pivotal role of fatty acid metabolism in the pathophysiology of asthma, we speculate that it may also play a key role in airway inflammation in RW. Elucidating the relationship between fatty acid metabolism and RW may improve our understanding of the pathogenesis of RW and facilitate the development of early prevention and treatment strategies.

We identified five hub genes shared by RW and SA from the nasal orange and tracheal plum consensus modules. Cystatin SN (encoded by CST1), cystatin SA (encoded by CST2), and cystatin S (encoded by CST4) are members of the type 2 cystatin protein superfamily  (58). Cystatins constitute a large group of evolutionarily related proteins that act as protease inhibitors of papain-like proteases  (58). Recent studies have implicated CST1, CST2, and CST4 in T2 airway inflammation. Notably, CST1, CST2, and CST4 are up-regulated by *in vitro* stimulation of human airway epithelial cells with IL-13  (37) and up-regulated in bronchial brushing samples from both mild and moderate adult asthmatics  (59). CST1 expression in nasal epithelial cells is up-regulated by thymic stromal lymphopoietin and IL-33, which reciprocally amplifies the release of these “alarmins”  (60). Intranasal treatment with CST1 induces the production of T2-associated cytokines (IL-4, IL-5, IL-13) and increases Th2 cell infiltration in murine sinonasal mucosa  (58). Supplementaryly, CST1 enhances eosinophil activation and recruitment by inducing IL-5 production in nasal polyp cells isolated from patients with eosinophilic chronic rhinosinusitis  (61). However, further work is necessary to clarify the specific functional roles of CST2 and CST4 in T2 airway inflammation. Brain-derived neurotrophic factor (BNDF) and its receptor, neurotrophic tyrosine kinase receptor type 2 (NTRK2), play important roles in neuronal differentiation, maturation, and survival  (62). In mammals, airway smooth muscle contractions are primarily mediated by parasympathetic cholinergic neurons  (63). One previous study  (64) has reported that mRNA expression of NTRK2 is elevated in bronchial biopsies of adult asthmatics and disruption of BDNF/NTRK2 signaling by NTRK2 receptor blockade down-regulates cholinergic innervation density in the airways of an ovalbumin-induced murine model of asthma, thereby ameliorating airway hyperresponsiveness. POSTN encodes for periostin, a secreted extracellular matrix protein that contributes to airway remodeling, a crucial pathophysiological feature of asthma  (65). POSTN is one of the most highly expressed genes in the airways of adult and school-age asthmatics  (35, 66). Furthermore, higher serum periostin levels in RW-impacted preschoolers are reported to be associated with an increased risk of acute wheezing attacks  (67) in the following year and risk of developing asthma at school-age  (68). All hub genes identified in this study are relevant to the pathogenesis of asthma, highlighting the need for follow-up studies to clarify their possible roles in the context of RW pathogenesis.

Finally, we found that the gene signatures of the five hub genes may be used to identify T2-high and T2-low subsets of RW. Previous studies have shown that POSTN and several other genes in airway epithelial brushings can be used to classify adult asthmatics into T2-high and T2-low endotypes  (35–37). Molecular endotyping of asthma based on T2 inflammation may have important clinical implications. First, compared to T2-low patients, T2-high patients exhibit a greater bronchodilatory response to salbutamol  (36) and significantly improved airway obstruction with inhaled steroids  (35). Therefore, classifying asthmatics based on T2 status may help to stratify patients for optimal treatment. Second, T2 gene expression is positively correlated with the degree of airway obstruction in adult asthmatics and is more pronounced in patients with poorly controlled asthma  (69). Therefore, evaluation of T2 status in asthma may contribute to better prognostic assessment. Over the last two decades, substantial efforts have been devoted to understanding the heterogeneity of preschool wheezing and tremendous advances have been made in defining wheezing phenotypes and understanding the longitudinal evolution of wheezing trajectories  (70). However, research exploring RW endotypes in disease subtypes with similar underlying pathophysiological mechanisms remains limited but could facilitate our understanding of RW pathogenesis and better targeted treatment. Interestingly, approximately 30% of RW patients were classified as T2-high based on hub gene expression (**Figures 2A–D**), which is lower than that reported for SA (over 80%)  (7). Thus, although the underlying mechanisms of RW and SA may overlap to some extent, mechanistic heterogeneity between them still exists. Large-scale prospective studies are needed to verify the roles of these hub genes in RW endotyping and to explore the significance of RW endotyping.

The present study has some limitations. First, the small sample size used for bioinformatics analysis may affect the robustness of the results. Second, as publicly available RW datasets are scarce, the extrapolation of our findings based on a single dataset may be limited, although the dataset contained high-quality RNA-seq data from both the upper and lower airway samples. Importantly, key results from transcriptomic analysis of nasal samples were well reproduced in parallel analysis of the tracheal transcriptome. Furthermore, the hub genes were validated by external datasets and qRT-PCR. Third, validation of hub genes was only performed at the transcriptional level, and further validation is required at both the protein and functional levels. Although our transcriptome analysis and external validation suggested that fatty acid metabolism-related pathways were possibly shared mechanisms for RW and SA, further in-depth studies are needed to verify this intriguing findings. Notably, although we integrated single-cell and bulk transcriptome datasets to estimate common changes in the cellular composition of airways in RW and SA, inferring cell composition with bulk transcriptome data may be less precise than using scRNA-seq. Finally, the raw data lacked corresponding clinical information, which may reveal new research perspectives when combined with our results.

In summary, co-expression network analysis revealed similarities in the transcriptome networks between RW and SA. Mast cells and club cells were identified as critical cellular components shared by RW and SA. Fatty acid metabolism-related biological processes and pathways were key signals in both RW and SA. Furthermore, five hub genes (CST1, CST2, CST4, POSTN, and NTRK2) were closely related to both RW and SA. Gene signatures of the five hub genes could be used to determine T2-high and T2-low subsets in RW patients. Collectively, these findings advance our understanding of the molecular pathogenesis of RW and provide a rationale for future exploration of the mechanistic relationship between RW and SA.

## Data availability statement

The original contributions presented in the study are included in the article/**Supplementary Material**. Further inquiries can be directed to the corresponding author.

## Ethics statement

The studies involving human participants were reviewed and approved by Ethics Committee of the Children’s Hospital of Chongqing Medical University, and written informed consent was obtained from the legal guardians of the study participants before enrollment.

## Author contributions

ZW conceived this study. YZ and QL, and ZG acquired data. ZW analyzed the data and performed the experiments. YZ, QL, and ZG visualized the data. ZW and YH interpreted the results. ZW and YH wrote the manuscript with all authors providing feedback for revision. ZL supervised the whole process. All authors contributed to the article and approved the submitted version.
