# Supplementary material for: Network analyses of upper and lower airway transcriptomes identify shared mechanisms among children with recurrent wheezing and school-age asthma
Source: Front Immunol. 2023 Jan 27;14:1087551. doi: 10.3389/fimmu.2023.1087551 (PMC9911682; doi:10.3389/fimmu.2023.1087551)

Supplementary Material

**Table S1. Demographic information for study participants**

| **Information** | **Controls** | **Recurrent wheezing** | **School-age asthma** |
| --- | --- | --- | --- |
| No. of participants | 18 | 32 | 15 |
| Age (y), mean (SD) | 4.1 (4.3) | 3.2 (1.5) | 7.9 (2.3) |
| Sex, male (%) | 61.1 | 59.3 | 53.3 |
| Past wheeze (%) | 14.3 | 100 | 100 |
| % Eosinophil, mean (SD) | 1.3 (0.9) | 1.9 (1.9) | 2.1 (1.2) |
| History of anaphylaxis (%) | 0 | 16.7 | 26.7 |
| Positive specific IgE (%) | NA | 33.3 | 40.0 |
| Eczema (%) | 21.4 | 29.1 | 46.7 |

IgE, immunoglobulin E; NA, not applicable; SD, standard deviation; y, year.

**Table S2. Data cohort characteristics.**

| **Dataset** | **RW(N)** | **SA (N)** | **HC (N)** | **Data type** | **Samples** | **Platform** | **Year** |
| --- | --- | --- | --- | --- | --- | --- | --- |
| GSE118761 | 14 | 13 | 14 | mRNA | Nasal/ tracheal  brushings | Illumina HiSeq 2000 | 2020 |
| GSE103166 | 56 | NA | 21 | mRNA | Nasal swabs | Affymetrix Human Gene 2.1 ST Array | 2019 |
| GSE65204 | NA | 36 | 33 | mRNA | Nasal  brushings | Agilent-028004 SurePrint G3 Human GE 8x60K Microarray | 2015 |
| GSE19187 | NA | 13 | 1 | mRNA | Nasal  brushings | Affymetrix Human Gene 1.0 ST Array | 2012 |
| FigShare.  14938755 | NA | NA | 18 | mRNA | Nasal cells | Illumina NovaSeq 6000 | 2021 |

HC, healthy control; NA, not applicable; RW, recurrent wheezing; SA, school-age asthma.

**Table S3. Primer sequence for qRT-PCR analysis**

| **Gene** | **Primers** | **Sequence (5’→3’)** |
| --- | --- | --- |
| CST1 | Forward primer | TACAGCGTGCCCTTCACTTC |
|  | Reverse primer | GTCTGTTGCCTGGCTCTTAGT |
| CST2 | Forward primer | GGAGGAGGACAGGATAATCGAGG |
|  | Reverse primer | ACAAAGTGAAGGGCACGCT |
| CST4 | Forward primer | TGTGTACCCTGCTACTCCTGA |
|  | Reverse primer | ACTCATCTTCGGTGGCCTTG |
| POSTN | Forward primer | AATCCCCGTGACTGTCTATAAGC |
|  | Reverse primer | TGTCTCCCTGAAGCAGTCTTTTAAT |
| NTRK2 | Forward primer | TCTGCTCACTTCATGGGCTG |
|  | Reverse primer | AGACCGAGAGATGTTCCCGA |
| GAPDH | Forward primer | GTCTCCTCTGACTTCAACAGCG |
|  | Reverse primer | ACCACCCTGTTGCTGTAGCCAA |

qRT-PCR, quantitative reverse transcription-polymerase chain reaction.

**Figure S1. Correspondence of RW-specific and SA-specific co-expressed gene network modules across nasal (A) and tracheal (B) transcriptomes.** Each row of the table corresponds to one RW-specific module (labeled by color as well as text), and each column corresponds to one SA-specific module. Numbers in the table indicate gene counts in the intersection of the corresponding modules. Coloring of the table encodes −log(*P*), with *P* being the Fisher’s exact test *P* value for the overlap of the two modules. The stronger the red color, the more significant the overlap is. The tables indicates that most RW-specific modules have one or more SA-specific modules counterpart. RW, recurrent wheezing; SA, school-age asthma.


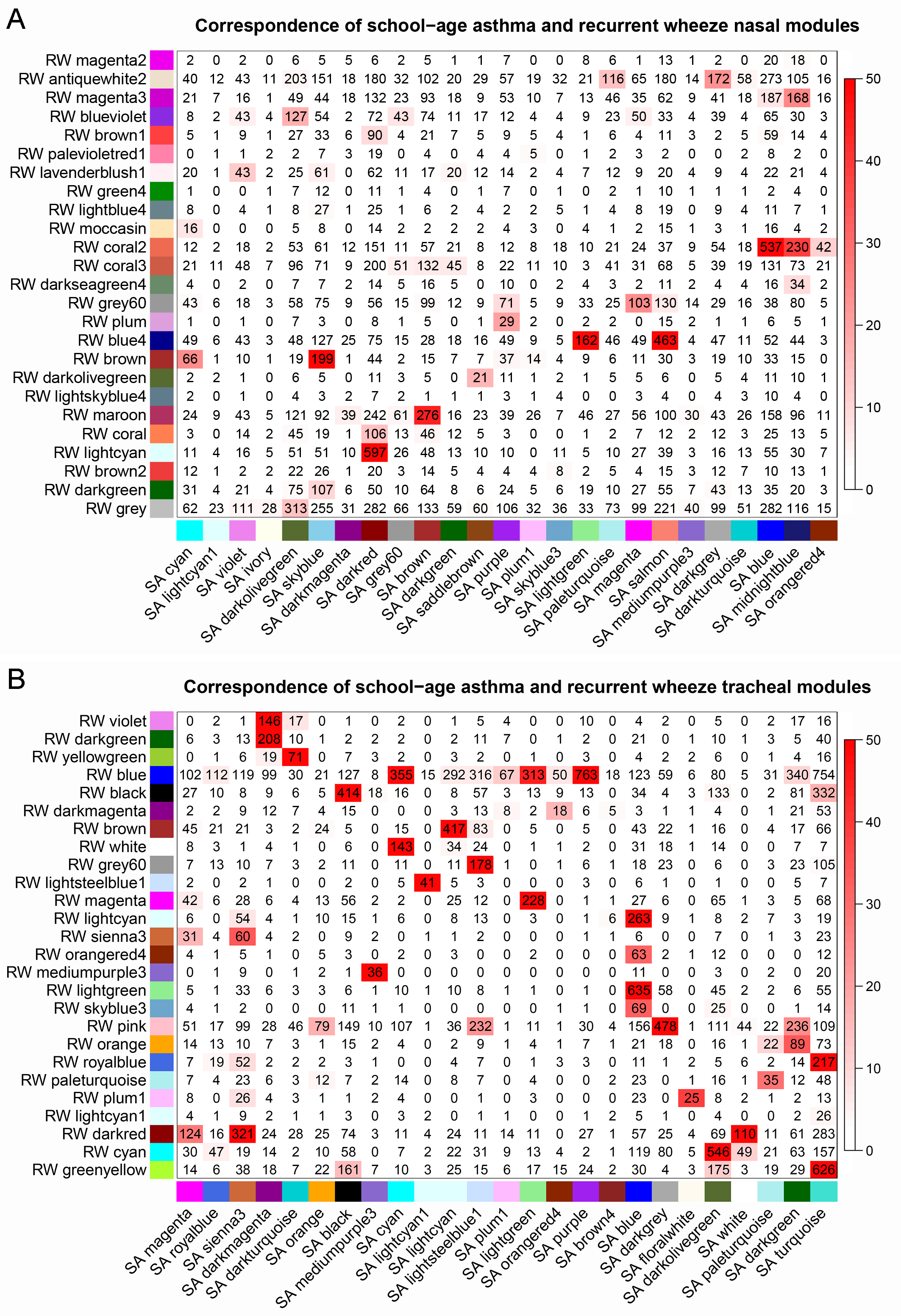


**Figure S2. Summary nasal consensus network indices (y-axes) as functions of the soft thresholding power (x-axes).** Numbers in the plots indicate the corresponding soft thresholding powers. The plots indicate that approximate scale-free topology is attained around the soft-thresholding power of 20 for the two sets. Because the summary connectivity measures (mean, max and median) decline steeply with increasing soft-thresholding power, it is advantageous to choose the lowest power that satisfies the approximate scale-free topology criterion.


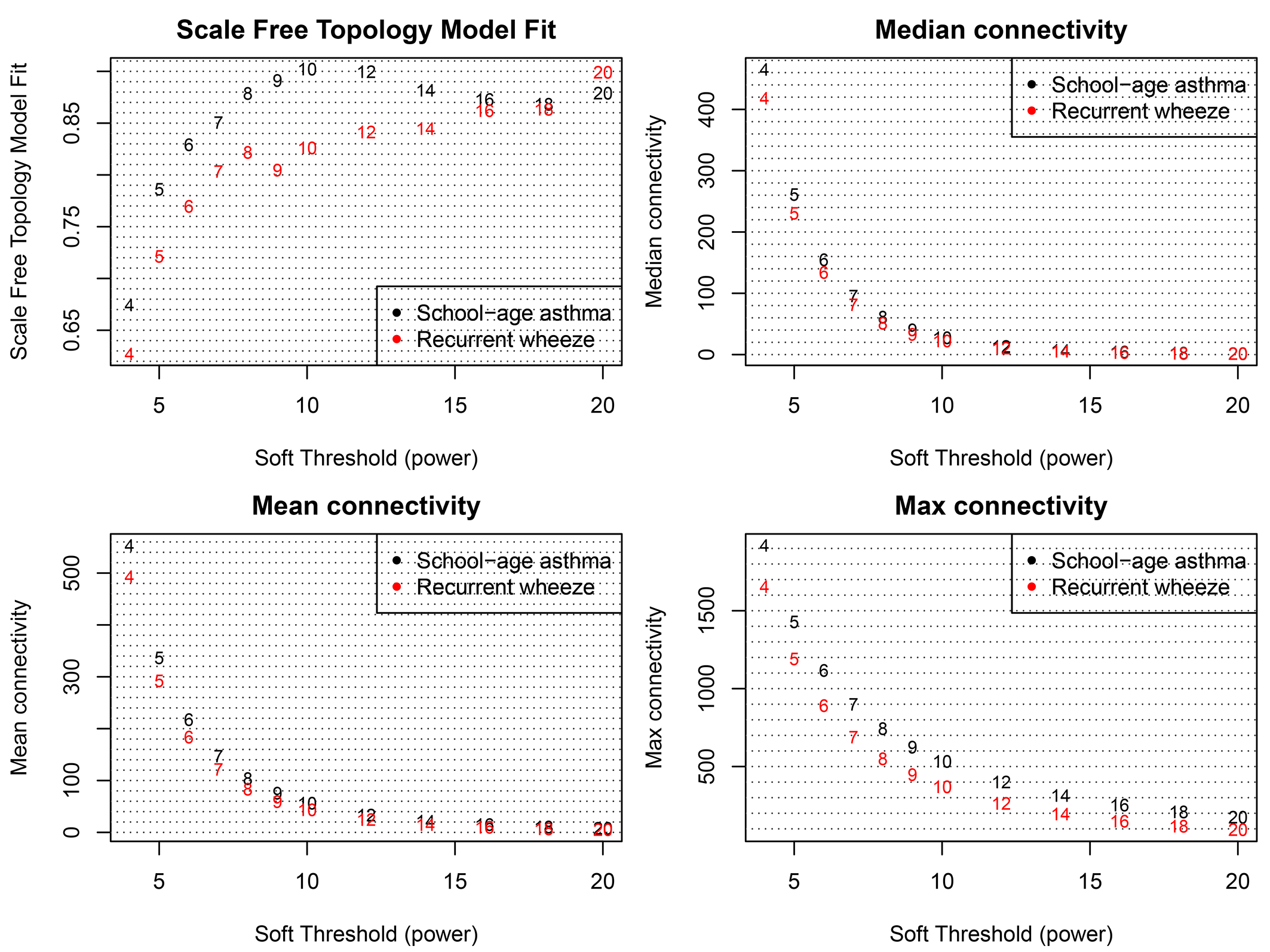


**Figure S3. Summary tracheal consensus network indices (y-axes) as functions of the soft thresholding power (x-axes).** Numbers in the plots indicate the corresponding soft thresholding powers. The plots indicate that approximate scale-free topology is attained around the soft-thresholding power of 16 for the two sets. Because the summary connectivity measures (mean, max and median) decline steeply with increasing soft-thresholding power, it is advantageous to choose the lowest power that satisfies the approximate scale-free topology criterion.


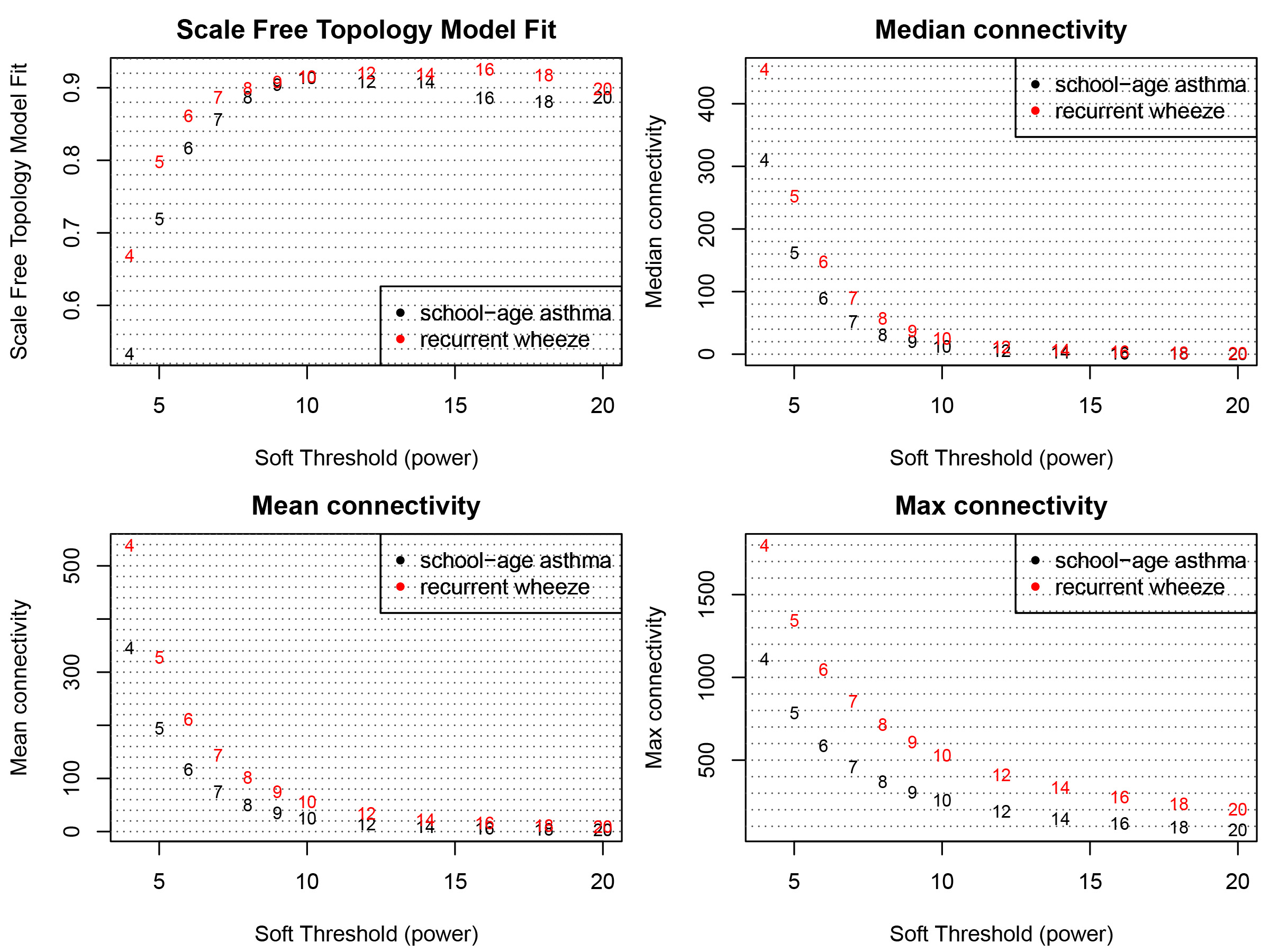


**Figure S4. Nasal (A) and tracheal (B) consensus weighted gene co-expression network analysis.** Modules were identified in the resulting dendrogram using the Dynamic Tree Cut algorithm. A total of 23 and 25 consensus modules were identified in nasal and tracheal samples seperately. Modules were distinguished from each other by assigning different colors.


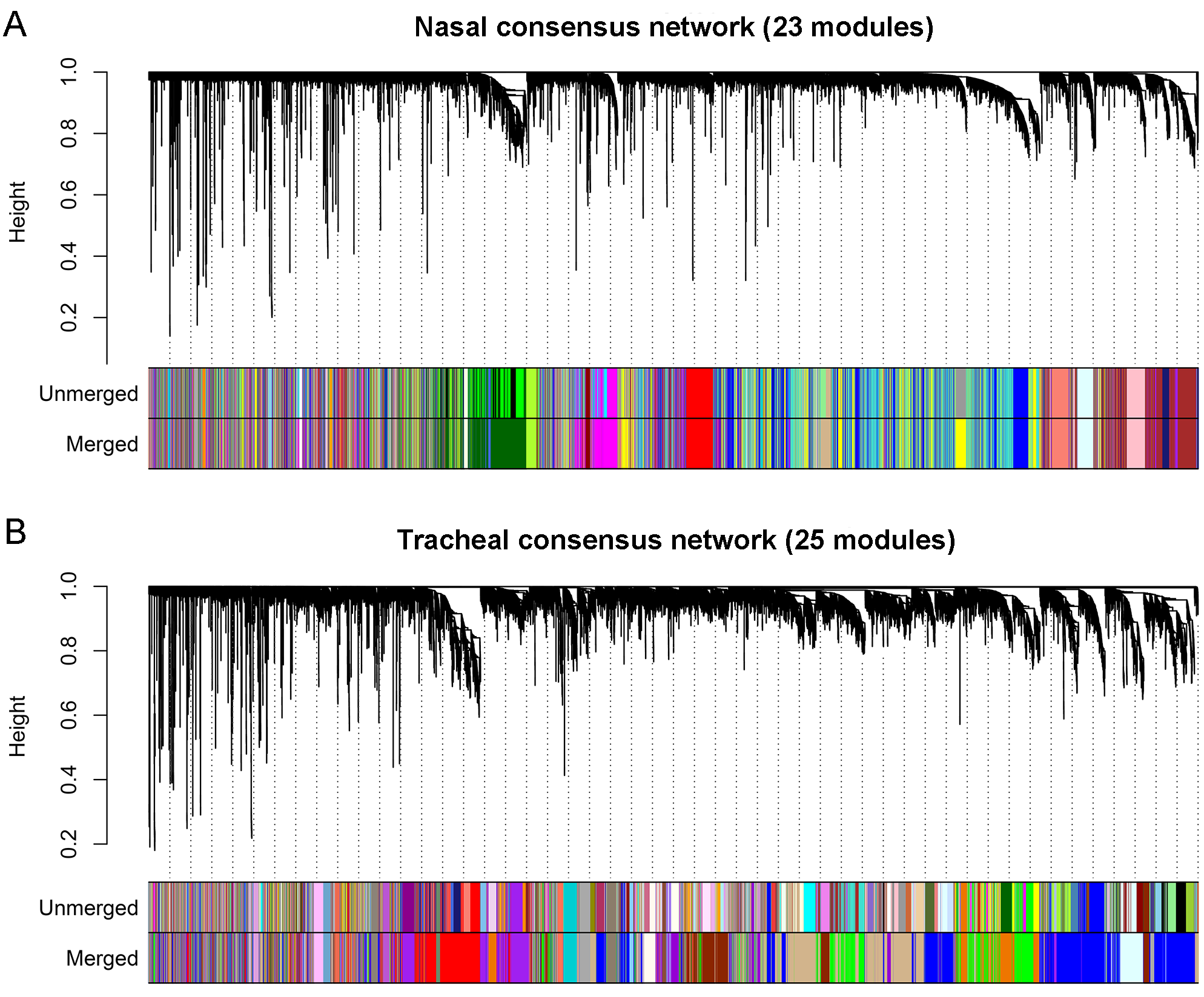


**Figure S5. Dotplot of cell type marker genes.** Dot plots depicting average and percent expression of genes used to classify **(A)** epithelial and **(B)** immune cells. The colour key from gray to blue indicated low to high expression levels. The dot size indicated the percentage of cells that expressed genes.


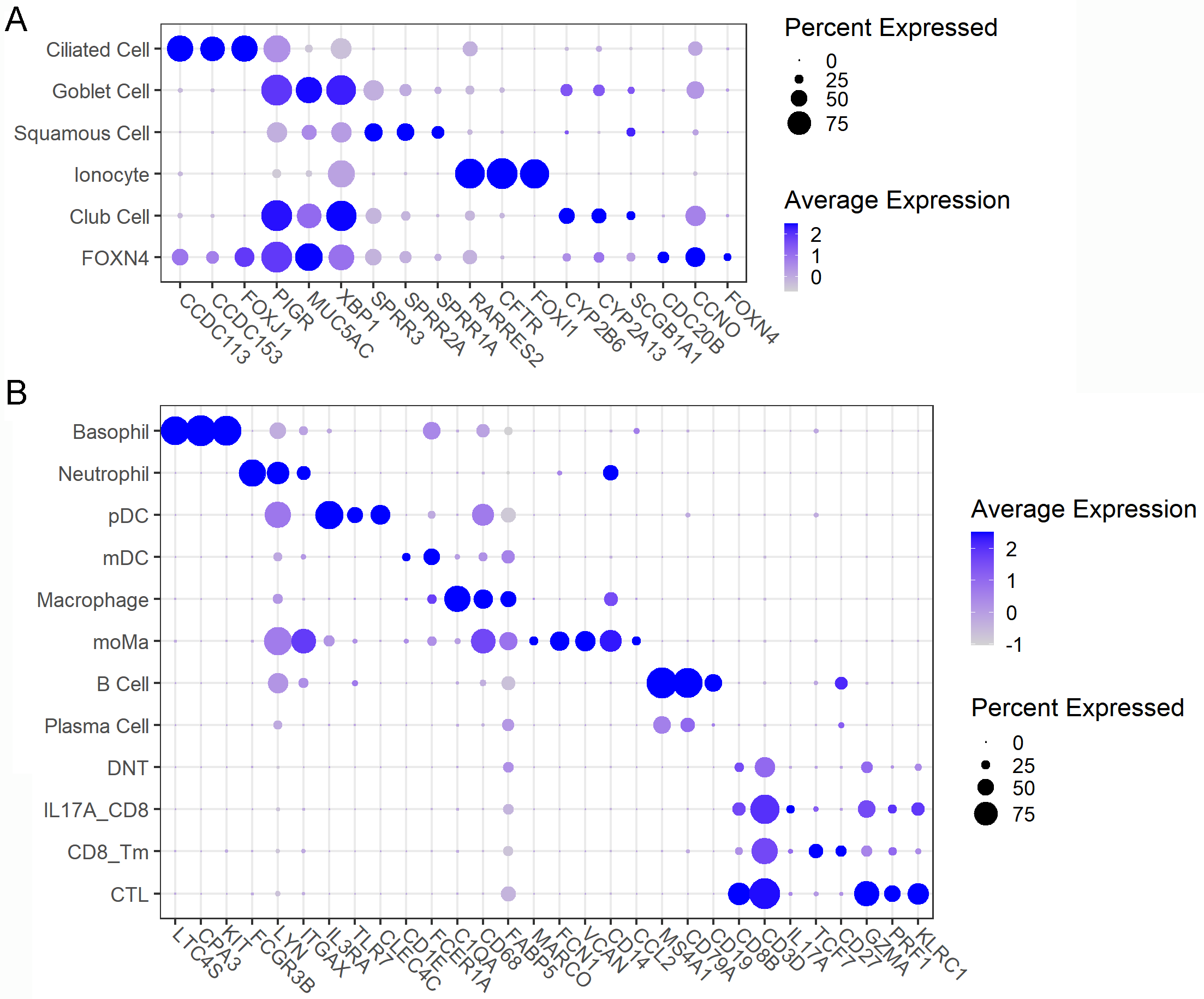


**Figure S6. Nasal consensus module relationships in recurrent wheezing (A) and school-age asthma (B).** Each module was represented by its eigengene value and Pearson correlation analyses between each of the eigengene values and clinical traits were performed. Each color represents one consensus gene module. Each row corresponds to a module eigengene, and column to a clinical trait. Each cell contains corresponding Pearson correlation coefficient (the first number) and *P* value (the number in the parenthesis). A green to purple gradient coloration implies an increased Pearson correlation coefficient.


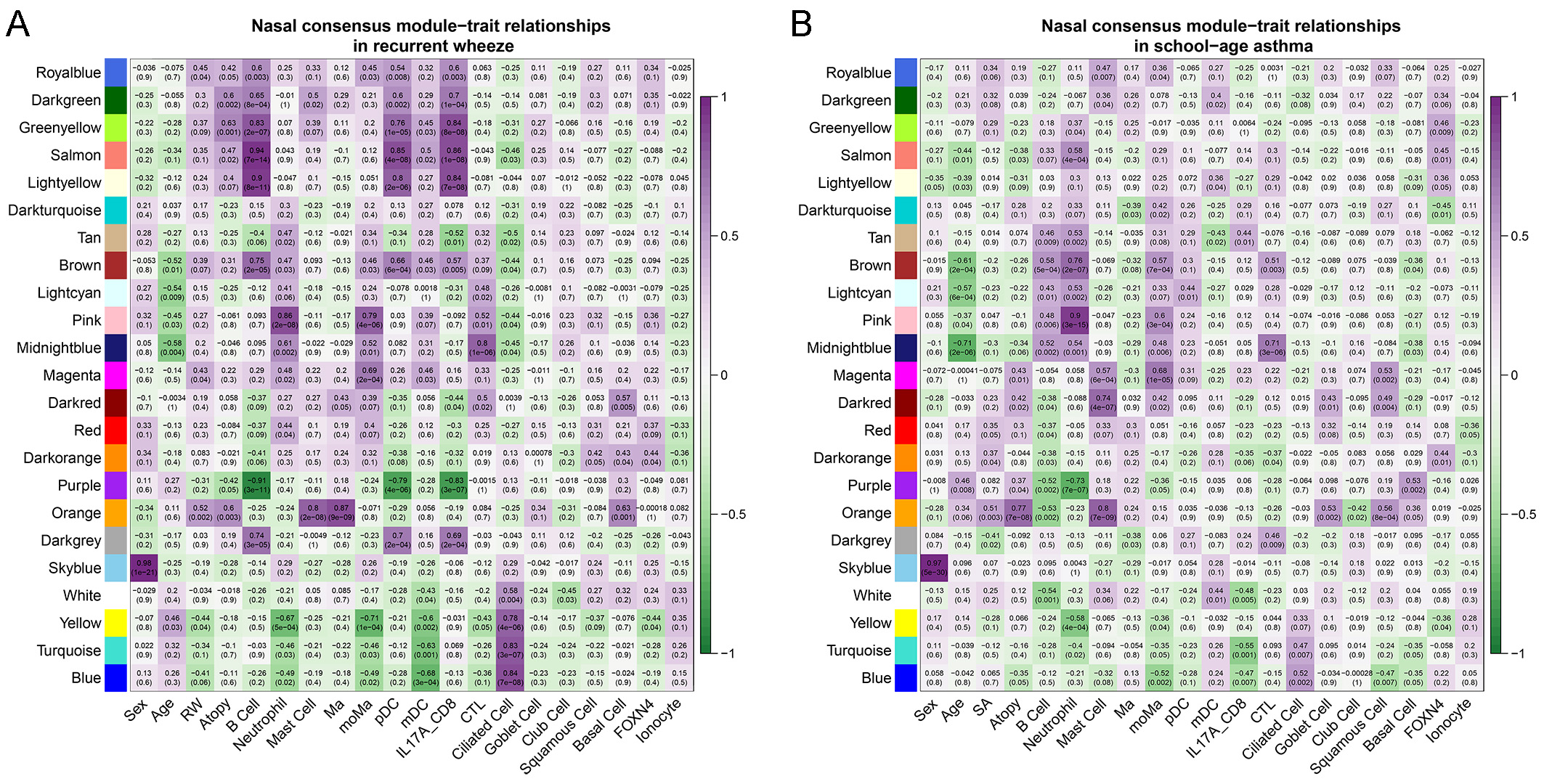


**Figure S7. Tracheal consensus module relationships in recurrent wheezing (A) and school-age asthma (B).** Each module was represented by its eigengene value and Pearson correlation analyses between each of the eigengene values and clinical traits were performed. Each color represents one consensus gene module. Each row corresponds to a module eigengene, and column to a clinical trait. Each cell contains corresponding Pearson correlation coefficient (the first number) and *P* value (the number in the parenthesis). A green to purple gradient coloration implies an increased Pearson correlation coefficient.


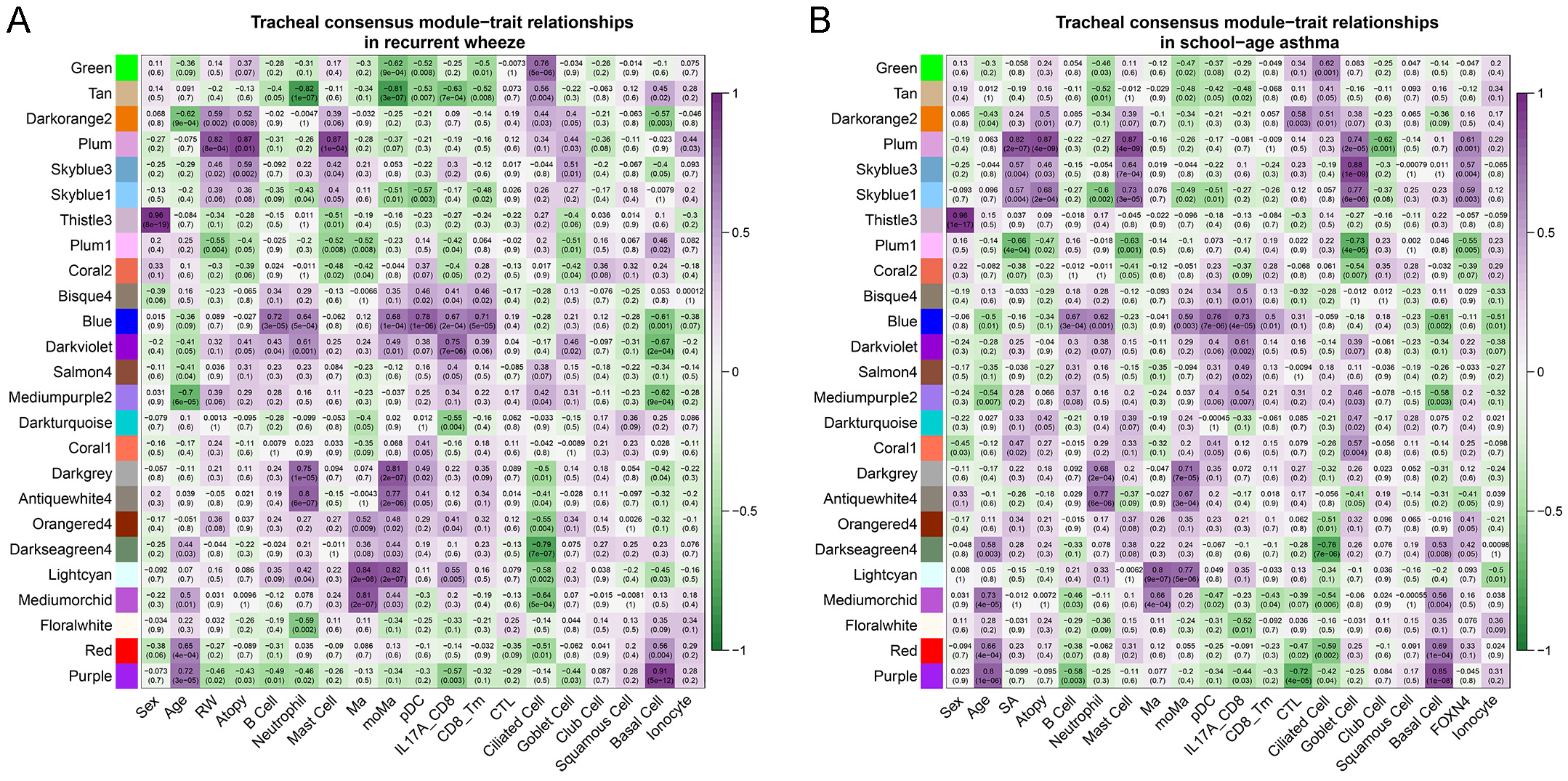


**Figure S8. Validation of shared mechanisms between RW and SA. (A)** Volcano plot showing differentially expressed genes (DEGs) between controls and children with SA in GSE65204 dataset. **(B)** Volcano plot showing DEGs between controls and patients with RW in GSE103166 dataset. **(C)** Venn diagram showing common DEGs between RW and SA. **(D)** Dot plot showing enriched GO terms (BP, CC, and MF) and KEGG pathways for common DEGs between RW and SA. BP, biological process; CC, cellular component; KEGG, Kyoto Encyclopedia of Genes and Genomes; MF, molecular function; RW, recurrent wheezing; SA, school-age asthma.


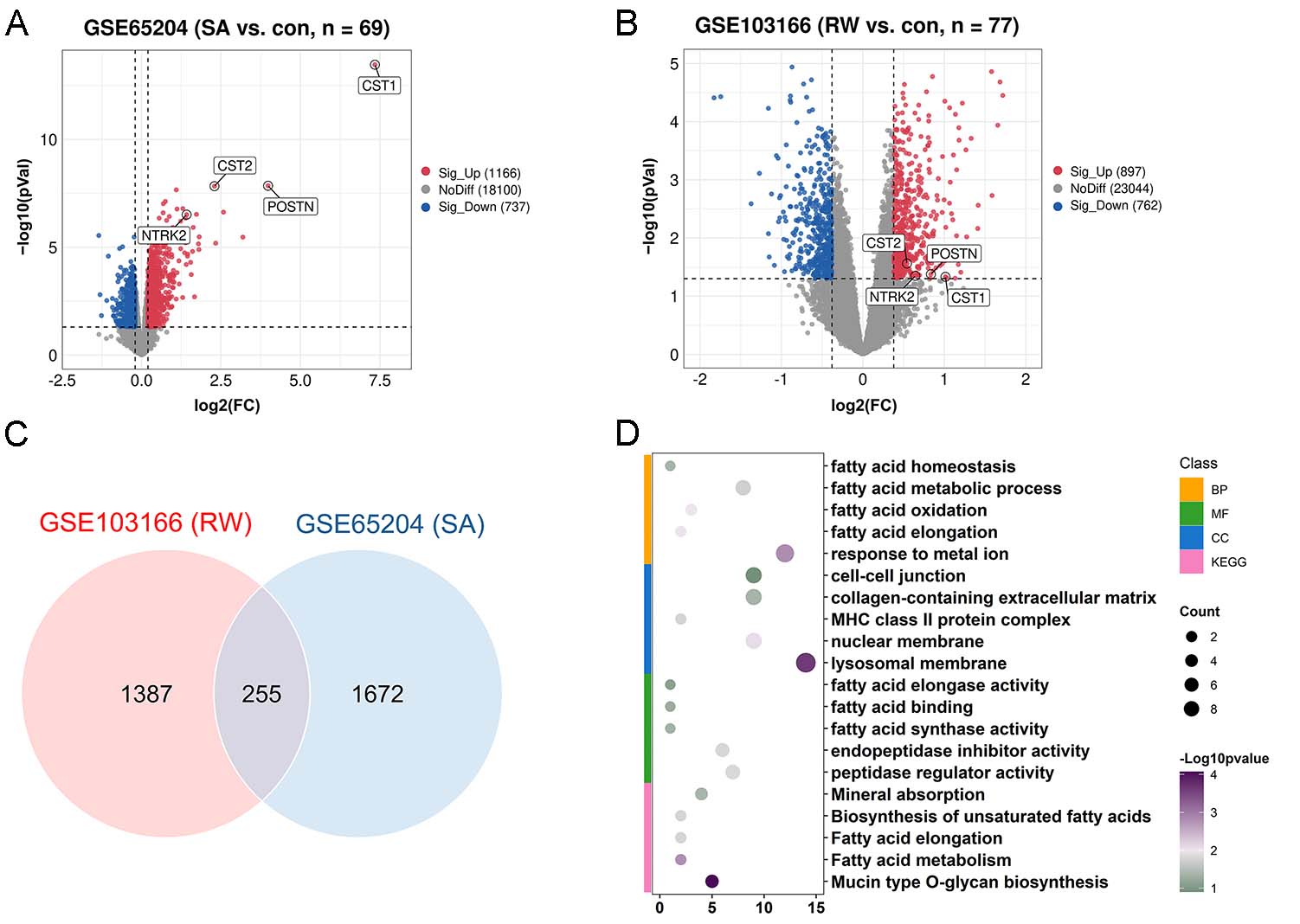


**Figure S9. Using hub genes could distinguish from RW/SA with control subjects. (A-D)** Discriminatory utility of the five hub genes across nasal and tracheal samples. Validation of expression levels of hub genes in GSE19187 **(E),** GSE65204 **(F)**, and GSE103166 **(G)**. CST4 was not detected in the gene expression profiling data of GSE65204 and GSE103166. Statistical significance was assessed using Wilcoxon rank-sum test. Asterisks indicated *P* values for SA or RW versus control. * *P* < 0.05, *** *P* < 0.001. Con, control; RW, recurrent wheezing; SA, school-age asthma.


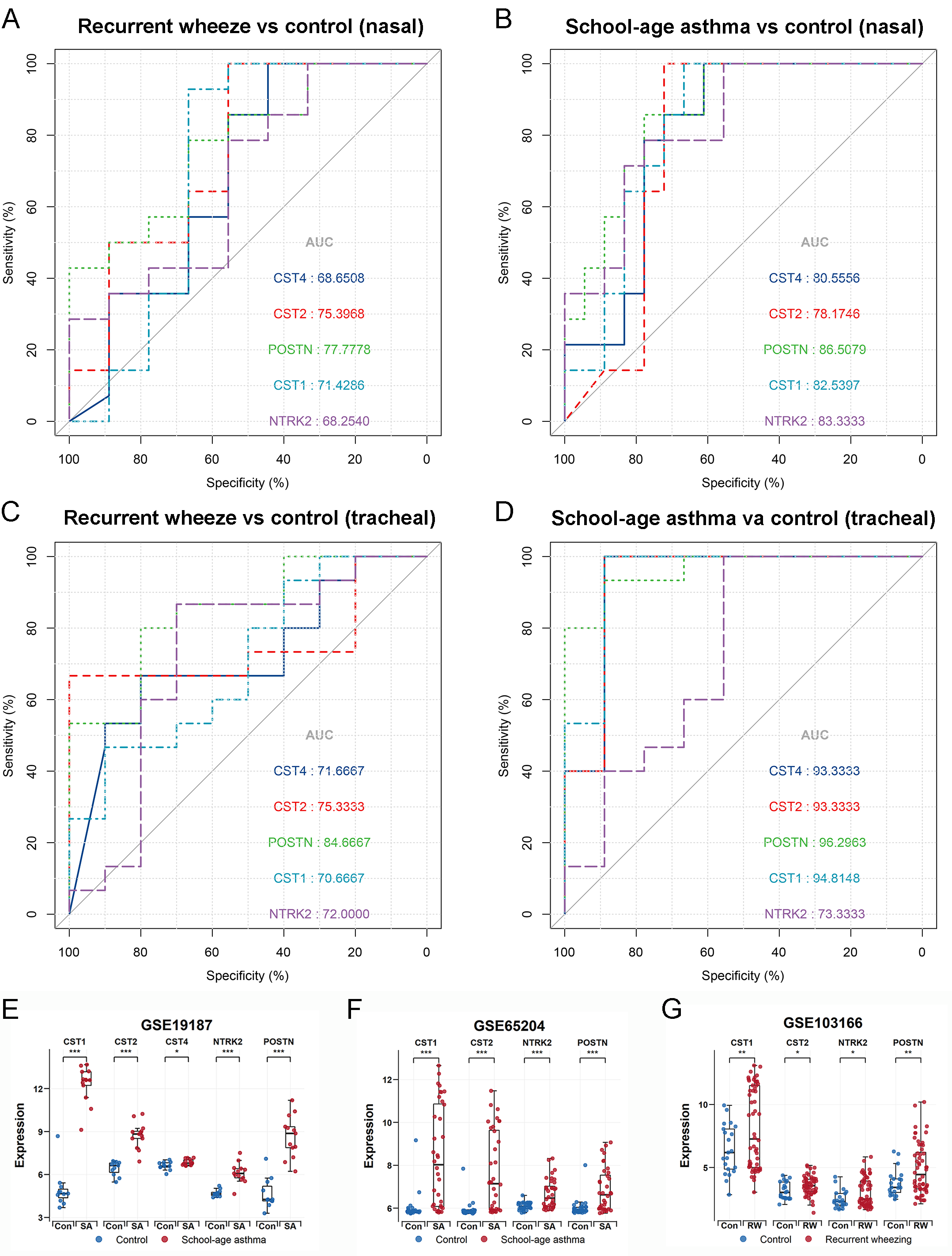


**Figure S10. TF-gene regulatory network of hub genes constructed using “RcisTarget” R package.** Square green represent five hub genes and circular nodes represent TFs. AUC, area under receiver operating characteristic (ROC) curve; NES, normalized enrichment score; TF, transcription factor.


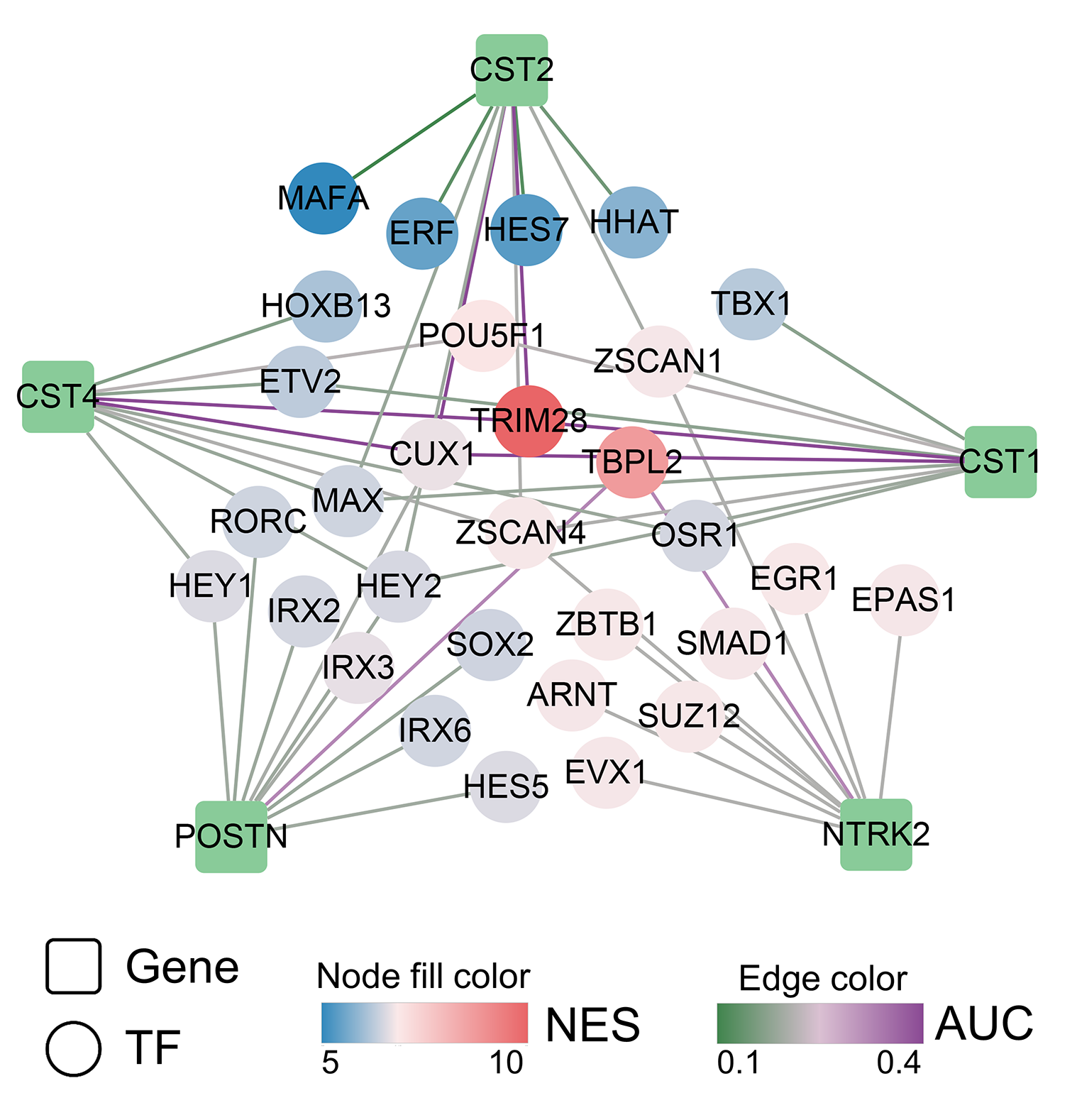

Supplement: Supplementary file 1 [file DataSheet_1.doc]
